# Human rhinovirus infection of human bronchial epithelial cells results in migration of human bronchial fibroblast cells

**DOI:** 10.1186/1710-1492-10-S1-A49

**Published:** 2014-03-03

**Authors:** Christopher Shelfoon, Sami Shariff, Suzanne Traves, Jason Arnason, Sergei Nikitenko, Richard Leigh, David Proud

**Affiliations:** 1 Snyder Institute for Chronic Disease, University of Calgary, Calgary, Alberta, T2N 4N1, Canada

## Background

Recent studies have demonstrated that structural changes in the airways characteristic of asthma, collectively referred to as airway remodeling, occur in young children even prior to the diagnosis of asthma. Young children who experience human rhinovirus (HRV)-associated wheezing illness within the first three years of life are at increased risk for the subsequent development of asthma. This association, together with evidence that HRV-infected epithelial cells release a number of growth factors and cytokines, has led to the hypothesis that HRV infection may be involved in the pathogenesis of airway remodeling. Thickening of the lamina reticularis found below the true basement membrane in the human airway is a characteristic feature of airway remodeling in asthma. The human bronchial fibroblast (HBF) is believed to contribute to this thickening by moving closer to the laminar reticularis and producing matrix proteins. We hypothesized that HRV infection of human bronchial epithelial (HBE) cells induces production of chemoattractants that can induce HBF migration.

## Methods

Primary HBE cells were grown in bronchial epithelial growth medium (BEGM). Cells were incubated for 2 h in bronchial epithelial basal media (BEBM) prior to experiments. Cells were exposed to BEBM alone (control) or purified HRV-16 (MOI: 1) for 24 hours. HBE cell supernatants from several donors were then recovered, centrifuged, pooled and studied as chemoattractants for HBF chemotaxis. HBF migration was examined using both a 48-well modified Boyden chamber and a 16-well xCELLigence^®^ apparatus (ACEA Biosciences, Inc., San Diego, USA). In the latter, migration to HBE supernatants was measured via electrical impedance, as per manufacturer’s instructions, and compared to medium control.

## Results

Using the xCELLigence system to measure migration in real time, it was found that migration to HBE supernatants and platelet derived growth factor (positive control) was maximal within 6h. Supernatants from HRV-16 infected HBE cells resulted in greater HBF migration compared to supernatants from HBE cells exposed to medium alone (negative control) in both the Boyden chamber and the xCELLigence system. Migration to supernatants from HRV-16 infected cells was concentration dependent.

## Conclusions

These data provide the first demonstration that HRV infection of HBE produces soluble factor(s) that cause migration of HBF cells. This provides further evidence for a potential role of HRV infection in the pathogenesis of airway remodeling in asthma.

